# Epigenetics in Liver Fibrosis: Could HDACs be a Therapeutic Target?

**DOI:** 10.3390/cells9102321

**Published:** 2020-10-19

**Authors:** Alex Claveria-Cabello, Leticia Colyn, Maria Arechederra, Jesus M. Urman, Carmen Berasain, Matias A. Avila, Maite G. Fernandez-Barrena

**Affiliations:** 1 Program of Hepatology, Center for Applied Medical Research (CIMA), University of Navarra, 31008 Pamplona, Spain; aclaveria.1@alumni.unav.es (A.C.-C.); lcolyn@alumni.unav.es (L.C.); macalderon@unav.es (M.A.); cberasain@unav.es (C.B.); 2 National Institute for the Study of Liver and Gastrointestinal Diseases (CIBERehd, Carlos III Health Institute), 28029 Madrid, Spain; 3 IdiSNA, Navarra Institute for Health Research, 31008 Pamplona, Spain; jm.urman.fernandez@navarra.es; 4 Department of Gastroenterology and Hepatology, Navarra University Hospital Complex, 31008 Pamplona, Spain

**Keywords:** liver fibrosis, epigenetics, histone deacetylases, precision medicine

## Abstract

Chronic liver diseases (CLD) represent a worldwide health problem. While CLDs may have diverse etiologies, a common pathogenic denominator is the presence of liver fibrosis. Cirrhosis, the end-stage of CLD, is characterized by extensive fibrosis and is markedly associated with the development of hepatocellular carcinoma. The most important event in hepatic fibrogenesis is the activation of hepatic stellate cells (HSC) following liver injury. Activated HSCs acquire a myofibroblast-like phenotype becoming proliferative, fibrogenic, and contractile cells. While transient activation of HSCs is part of the physiological mechanisms of tissue repair, protracted activation of a wound healing reaction leads to organ fibrosis. The phenotypic changes of activated HSCs involve epigenetic mechanisms mediated by non-coding RNAs (ncRNA) as well as by changes in DNA methylation and histone modifications. During CLD these epigenetic mechanisms become deregulated, with alterations in the expression and activity of epigenetic modulators. Here we provide an overview of the epigenetic alterations involved in fibrogenic HSCs transdifferentiation with particular focus on histones acetylation changes. We also discuss recent studies supporting the promising therapeutic potential of histone deacetylase inhibitors in liver fibrosis.

## 1. Introduction

### 1.1. Liver Fibrosis

Chronic liver disease (CLD) encompasses many different etiologies, from viral infections and auto-immune conditions, to alcohol abuse and metabolic disorders (i.e., non-alcoholic steatohepatitis, NASH). All of them converge in liver fibrosis, which is a common endpoint of almost every CLD [1,2]. Liver fibrosis is defined as the excessive accumulation of fibrous connective tissue in and around an injured area of the liver [1]. Fibrogenesis is normally a wound healing response to an acute or transient injury to an organ, which architecture is restored afterwards. However, when the injury is perpetuated as in CLD, this wound healing response is sustained and leads to the accumulation of extracellular matrix (ECM), an event known as hepatic fibrogenesis [3,4,5]. The fibrosis end-phase is known as cirrhosis, which involves the substitution of liver parenchyma for scar tissue, the alteration of the organ’s architecture and the impairment of liver function due in part to the dedifferentiation of hepatocytes [6,7]. Cirrhosis markedly increases the chances of developing hepatocellular carcinoma (HCC), the most frequent type of liver cancer [3,5,8]. From 1990 to 2017 cirrhosis-caused deaths increased from less than 900,000 to more than 1.32 million per year globally. Importantly, the prevalence of cirrhosis caused by NASH increased more than any other cause in that period of time [9].

The ECM is produced by myofibroblasts that can have different origins: portal fibroblasts [10,11], bone marrow [12,13], fibrocytes [14], and hepatic stellate cells (HSCs) which are considered to be their major source [3,14,15]. In normal liver, HSCs are perisinusoidal cells located in the space of Disse that represent the most important vitamin A storage compartment in the organism [5]. These cells have other tasks in normal liver such as the control of ECM turnover and the regulation of sinusoids contractility [15]. Following liver injury, HSCs become activated and undergo transdifferentiation to ECM-producing myofibroblasts. HSCs activation remains the most important pathway for hepatic fibrogenesis [16]. In this event, HSCs lose their retinoid droplets and become proliferative, fibrogenic and contractile cells [15]. Importantly, after cessation of injury activated HSCs may regress to an inactive phenotype similar to their quiescent state but still they remain more responsive to fibrogenic stimuli [17,18]. These rapid phenotypic modifications involve considerable gene expression changes [3,15], which are in part governed by epigenetic mechanisms. Therefore, epigenetics are certainly critical in the HSCs activation process [1].

“Epigenetics” refers to reversible and inheritable changes in gene expression that do not implicate modifications to the underlying DNA sequence [19]. Epigenetic mechanisms are to a great extent responsible for the establishment and maintenance of the different cellular identities emerging from a common genome in multicellular organisms [1]. It is now accepted that the transdifferentiation of HSCs requires a global epigenetic adjustment to silence adipogenic differentiation factors and enhance de novo expression of genes associated with the new phenotype [3,20]. In this review, we summarize the current knowledge about the relationship between epigenetics and liver fibrosis, with special focus on the acetylation and deacetylation of histones.

### 1.2. Epigenetic Mechanisms

The epigenetic mechanisms are mediated at least by three processes: the expression of non-coding RNAs (ncRNAs), DNA methylation and histone modifications [5]. Chromatin configuration is the result of the crosstalk between these overlapping and interacting mechanisms [1]. Furthermore, histone modifications include at least 8 different covalent changes that take place in the N-terminal tails of the histones, and the best-characterized are acetylation (lysine), methylation (lysine/arginine) and phosphorylation (threonine/serine) [21].

#### 1.2.1. Non-coding RNAs

Within the whole human genome only 2% encodes proteins. The majority of the transcriptome comprises non-coding RNAs (ncRNAs) [1]. ncRNAs exert regulatory functions and include micro RNAs (miRNA), small nucleolar RNAs (snoRNAs), small interferent RNAs (siRNAs), Piwi-interacting RNAs (piRNAs) and long non-coding RNAs (lncRNAs) [22]. The most characterized ncRNAs are the miRNAs which are single-stranded 18–22 nucleotide ncRNAs that repress gene expression by decreasing stability or inhibiting translation of messenger RNAs [22]. LncRNAs are >200 nucleotides long, and are involved in the regulation of several biological processes such as cell proliferation, differentiation, migration and survival, even though their mechanisms of action are not yet fully described [23].

#### 1.2.2. DNA Methylation

DNA methylation is the best-characterized epigenetic event [23]. In this process the carbon atom in position five of the cytosine residues within cytosine-phospho-guanine (CpG) dinucleotides is methylated. Most human gene promoters contain one or more large regions enriched in CpG dinucleotides (>55%) called CpG islands, which are normally found in an unmethylated state [5,23]. Methylation in CpG islands is mainly associated with gene repression. This occurs by two mechanisms: methylation can condense chromatin avoiding the binding of transcription factors, and CpG methylation can mediate the recruitment of methyl-binding proteins (MeCP2, MBD1–4) that cooperate with chromatin silencing complexes [3,5,23]. However, DNA methylation may also have other effects, as it has been described to activate gene transcription when low-methylated CpG islands become hypermethylated or when methylation occurs within gene bodies [1,3]. This epigenetic modification is carried out by DNA methyltransferases (DNMTs) that are divided in two main classes: the enzymes in charge of de novo DNA methylation (DNMT3A and DNMT3B) and that implicated in the conservation of the postreplicative DNA methylation pattern (DNMT1) [3,23]. Nevertheless, accumulating evidence indicate that both types can display both types of activities depending on the DNA sequence context [24].

Global methylation levels are also regulated by the DNA demethylation process carried out by the Ten Eleven Translocation (TET) enzyme family, including TET1, TET2, and TET3. These enzymes oxidize the 5-methylcytosine (5mC) residue to 5-hydroxymethylcytosine (5hmC), 5-formylcytosine (5fC) and 5-carboxylcytosine (5caC) in an active process [25]. These oxidized forms of 5mC can be removed in a two-step process involving the sequential action of thymine-DNA-glycosylase (TDG) coupled with base excision repair (BER) [26].

#### 1.2.3. Histone Methylation

The outcome of histone methylation depends on the precise location of the lysine residue on the histone tail and on the extent of its methylation [3,22]. For instance, trimethylation of lysine 9 of histone 3 (H3K9me3) and H3K27me3 are related to transcriptional silencing. On the other hand, H3K4me3, H3K36me2/3, and H3K79me3 are associated with active gene transcription [5,23]. The introduction of methyl groups in lysine or arginine residues on histone tails is carried out by histone methyltransferases (HMTs), whereas demethylation implicates histone demethylases (HDMTs) [3].

#### 1.2.4. Histone Acetylation/Deacetylation

Histones are enriched in lysine and arginine residues which provide them with strong basic properties. These positively charged residues facilitate the interaction with the negatively charged DNA. The introduction of acetyl groups by histone acetyltransferases (HATs) neutralizes the positive charge of lysines and decreases the affinity of histones for DNA. This results in a relaxed chromatin conformation, which allows the access of transcription factors and the transcriptional machinery which drive gene expression. On the other hand, histone deacetylation, carried out by histone deacetylases (HDACs), leads to chromatin compaction, preventing the binding of transcription factors and resulting in gene repression [27,28,29]. Therefore, HDACs generally have the opposite function to HATs, and both of them regulate the histone acetylation code in a reversible process [30,31].

To date, 18 HDACs have been identified in mammals. HDACs are divided into two categories and four subclasses (Table 1), depending on sequence homology to yeast HDACs and domain organization: the zinc-dependent HDACs (Class I, II, and IV) and the nicotinamide adenine dinucleotide (NAD^+^)-dependent HDACs (Class III) [27,29]. Class I HDACs (HDAC1, 2, 3, and 8) are ubiquitously expressed and almost completely located in the nucleus. They share high homology in their catalytic sites with yeast histone deacetylase RPD3 [29,31,32]. Class II HDACs is subcategorised in Class IIa (HDACs 4, 5, 7, and 9) with just one catalytic site, and Class IIb (HDACs 6 and 10) with two catalytic sites. Unlike Class I HDACs, they are expressed in a tissue-specific manner in mammals and mainly located to the cytoplasm but can shuttle in and out of the nucleus in response to cellular signals [29,30]. Class II HDACs are closely related to yeast HDA1 [29,32]. Class III HDACs, also called sirtuins, are structurally and functionally different from the rest of the HDACs, as they require NAD^+^ as a cofactor [29,30,31]. This group includes seven sirtuins (SIRT1, 2, 3, 4, 5, 6, and 7) which have an interesting subcellular location: SIRT1 and SIRT2 localize to the nucleus and cytoplasm, SIRT3 to the nucleus and mitochondria, SIRT4 and SIRT5 only in the mitochondria, SIRT6 exclusively in the nucleus and SIRT7 in the nucleolus [29,31]. Finally, HDAC11 is the only representative of Class IV HDACs, localizing to the nucleus and sharing sequence homology with Class I and Class II HDACs [31,32]. HDACs are also called lysine deacetylases (KDACs). This is due to the fact that various non-histone proteins, such as transcription factors and tubulin, can be deacetylated by HDACs, resulting in the modification of their function and the regulation of different cellular processes [29,30,32].

### 1.3. Role of the Epigenetic Mechanisms in Liver Fibrosis

#### 1.3.1. Non-Coding RNAs

In liver fibrosis, the most studied ncRNAs are the miRNAs. Even though their biological function is less known, there are numerous studies showing the differential expression of several miRNAs in this condition [23]. One of them showed a downregulation of the miR-29 family members in experimental liver fibrosis and also in human fibrotic liver tissues and serum from patients at different stages of chronic liver disease [33]. Moreover, miR-29 downregulation was promoted by TGFβ-induced profibrogenic signals as well as by bacterial lipopolysaccharide (LPS)-induced inflammatory responses in HSC. Indeed, overexpression of miR-29 in murine HSC reduced collagen expression [33]. There are other miRNAs involved in the regulation of HSC activation which expression is also altered in this process [5,34]. For instance, microarray studies showed 12 upregulated miRNAs (miR-874, miR-29C, miR-501, miR-349, miR-325-5p, miR-328, miR-138, miR-143, miR-207, miR-872, miR-140, and miR-193) and nine downregulated miRNAs (miR-341, miR20b-3p, miR-15b, miR-16, miR-375, miR-122, miR-146a, miR-92b, and miR-126) during activation of hepatic stellate cells in a CCl_4_- induced liver fibrosis rat model [35]. The authors also proposed 25 pathways probably regulated by these miRNAs [35]. Nonetheless, additional miRNAs seem to be involved in the fibrogenic activation of HSCs including miR-181 [36,37], miR-125b [38], and miR-214 [39] among others (see Review [34]). Interestingly, other miRNAs exert an inhibitory function towards liver fibrosis, such as miR-455-3p [40] and miR-29 [33,41,42,43].

Although lncRNAs constitute more than 60% of the non-coding transcriptome their biological functions and mechanisms of action are still not well-known [44]. One of the first-described lncRNAs is H19, which is highly conserved in mammals. It seems to have an important role in tissue development as it shows high levels of expression during embryogenesis and decreases after birth in most tissues. However, H19 is overexpressed in several diseases including malignant digestive cancers [44]. The role of H19 in liver fibrosis remains controversial. A study showed that H19 expression was reduced in activated HSCs and in liver tissues from CCl_4_-treated rats, in association with increased DNMT1 expression and H19 promoter methylation levels [45]. On the contrary, a more recent study demonstrated that H19 overexpression in the liver promoted liver fibrosis in the bile duct ligation (BDL) model, Mdr2^-/-^ mice and upon CCl_4_ treatment in mice. Conversely, H19 deficiency protected from liver fibrosis induced by BDL and in Mdr2^-/-^ mice. Moreover, H19-enriched exosomes intensified the activation of cultured mouse primary HSCs and induced proliferation and ECM production in HSC-derived fibroblasts [46]. These data suggest a potential role of H19 in liver fibrosis, nevertheless further studies are needed to better understand the overall function of lncRNAs in HSCs activation.

#### 1.3.2. DNA Methylation

Liver fibrosis is associated with changes in the DNA methylation patterns and the expression and activity of the involved epigenetic enzymes [3,5,22]. A genome-wide analysis of DNA methylation status in CCl_4_ mouse liver tissues showed hypomethylation of genes linked to fibrosis development preceding the onset of liver fibrosis [47]. Moreover, a loss of almost 60% of the original DNA methylation levels has been reported to occur in rat primary HSC within the first three days of culture in serum-containing media on a plastic surface, a condition that leads to their fibrogenic activation [48]. Although HSC transdifferentiation into myofibroblasts is characterized by a general loss of DNA methylation, gene-specific DNA hypo- and hypermethylation correlating with altered gene expression has been confirmed in genome-wide DNA methylation analyses [48,49]. For instance, upregulation of profibrogenic genes such as *Actg2*, *Loxl1*, *Loxl2*, and *Col4A1/2* is associated with a decrease in promoter methylation levels in activated HSCs [49]. On the other hand, gene expression downregulation characterized by DNA hypermethylation was found in genes like *Smad7*, an antagonist of TGFβR1 signaling [50], and *Pten*, which is a negative regulator of HSC activation [51].

Recently, a study reported TET2 and TET3 downregulation in liver tissues from BDL-induced experimental fibrosis, which was accompanied by loss of 5hmC. In contrast, the protein levels of DNMTs were overall enhanced in fibrotic livers. These results correlated with global epigenetic changes in human fibrotic liver, showing low levels of 5hmC and upregulated expression of DNMT3A/B [52]. Hence, DNMTs/TETs expression changes are associated with genome-wide alterations in DNA methylation underlying HSC transdifferentiation [52]. In agreement to these studies, Mann et al. demonstrated that the DNMTs inhibitor 5-aza-2′-deoxycytidine (5-azadC) blocked rat primary HSC transdifferentiation preventing the acquisition of proinflammatory and profibrogenic traits [53].

As previously explained, DNA methylation is regulated in part by the attachment of methyl-binding proteins, which consecutively recruit transcriptional repressor complexes [1,5]. One of these methyl binding protein is MeCP2, whose expression is induced during the transdifferentiation of HSCs [1]. Indeed, MeCP2-deficient mice showed reduced liver fibrosis after CCl_4_ treatment as well as attenuated expression of fibrogenic markers like collagen-1, TIMP-1, and α-SMA [54]. Earlier studies showed that *PPARγ* expression must be silenced in HSC in order to become activated HSC and develop the myofibroblastic phenotype [55]. In line with this, it was demonstrated that MeCP2 repressed *PPARγ* expression through two different mechanisms. First, MeCP2 binds to methyl-CpG residues in the *PPARγ* promoter leading to the recruitment of epigenetic enzymes that silence gene transcription through H3K9me3 modification. Second, MeCP2 enhances the expression of EZH2, which binds to the downstream coding region of the *PPARγ* gene resulting in the inhibition of transcriptional elongation through H3K27me3 modifications [54]. Although MeCP2 can repress transcription from methylated gene promoters, it is also associated with active transcription from methylated regions [1,23]. For instance, MeCP2 induces the expression of ASH1, a histone methyltransferase that induces transcriptional activation of profibrogenic genes via H3K4 methylation [56]. Furthermore, a very recent study demonstrated that HIPK2-mediated Ser80 phosphorylation of MeCP2 is required for HSC transdifferentiation [57]. Mutation of Ser80 to alanine reduces the affinity of MeCP2 to multiple gene promoter sequences, therefore this post-translational modification is also important for DNA binding [58]. This finding identifies new potential therapeutic targets for antifibrogenic therapies.

#### 1.3.3. Histone Methylation

Several studies have proposed a crucial role for HMTs and HDMTs in liver fibrosis. For instance, MeCP2-mediated transdifferentiation of HSCs is in part orchestrated by two different histone methyltransferases: EZH2 and ASH1 [54,56]. This mechanism is one example that highlights the importance of the extensive crosstalk taking place among epigenetic modifications. As previously mentioned, EZH2 and ASH1 expression is induced during HSCs activation and, as a result, they enhance H3K27me3 and H3K4me modifications, respectively. Indeed, overexpression of EZH2 induces the expression of fibronectin, α-SMA and collagen 1α1 in primary human HSCs [59]. In fact, various studies showed that EZH2 inhibitors such as 3-deazaneplanocin A (DZNep) and GSK-503 had antifibrotic properties in in vitro models [54,59] as well as in in vivo models like CCl_4_ and BDL-induced liver fibrosis [59,60,61]. Mechanistically, a recent study demonstrated that EZH2-mediated suppression of *Dkk1* expression, a negative regulator of the Wnt/β-catenin pathway, which is required for HSCs activation [61]. This is important taking into account that Wnt/β-catenin pathway activation is necessary for HSCs transdifferentiation [62,63]. These studies suggest HMTs as interesting targets for therapeutic approaches in liver fibrosis.

Nevertheless, there are other HMTs involved in the pathogenesis of liver fibrosis. It has been demonstrated that ethanol induces the expression of MLL1, a H3K4 methyltransferase that binds to the *Elastin* gene promoter, a fibrosis-related protein. As a consequence, the promoter is enriched in H3K4me3, a mark of transcriptional activation [64]. In line with this, TGFβ-stimulated mouse embryonic fibroblasts (MEFs) and HSC-T6 (a rat cell line of HSCs) showed increased levels of H3K4me2 and H3K4me3 in the promoter of profibrogenic genes such as *COL1A1/2* and *α-SMA*. This process is mediated by a protein complex called COMPASS, which includes HMTs such as ASH2, SET1, and WDR5 [65]. Finally, our group recently demonstrated that, together with DNMT1, the HMT G9a is overexpressed in cirrhotic human liver, in BDL and CCl_4_-induced fibrosis in mice, and in activated primary mouse HSCs [66]. Furthermore, we showed that DNMT1/G9a dual inhibition has antifibrogenic activity in TGFβ-stimulated primary human HSC and in human precision-cut liver slices, an ex vivo model that preserves the cellular and structural characteristics of the liver parenchyma. The antifibrogenic potential of dual G9a/DNMT1 inhibition was also observed in relevant in vivo mouse models of liver fibrosis [66].

In addition to HMTs, HDMTs are also implicated in HSCs transdifferentiation. As an example, Domain-Containing Protein 1A (JMJD1A) is a HDMT involved in the regulation of PPARγ expression in HSCs. In the quiescent state, JMJD1A epigenetically modifies H3K9me2 levels on the *PPARγ* gene promoter, inducing its expression. In agreement with this, JMJD1A overexpression attenuated the myofibroblastic phenotype of primary rat HSCs. In contrast, JMJD1A knockdown correlated with increased H3K9me2 levels and downregulation of *PPARγ* gene expression [67]. Moreover, another HDMT, known as KDM4D, is upregulated in HSC activation. Consistently, in vivo KDM4D knockdown blocked fibrosis progression and promoted fibrosis reversal in the CCl_4_ mouse model. Mechanistically, this enzyme catalyzes H3K9me2/3 demethylation in the TLR4 gene promoter, thus activating the TLR4/NF-κB signaling pathway in HSCs and contributing to their activation [68]. KDM4A, KDM4B, and KDM4C, which are members of the same family of HDMTs but structurally different from KDM4D, are markedly downregulated during HSC activation. These three KDM4 enzymes regulate the expression of miR-29, an antagonist of liver fibrosis, during the activation of HSCs. In fact, KDM4A-C knockdown was followed by repression of miR-29 expression in vitro and in vivo [69].

## 2. Acetylation/Deacetylation of Histones in Liver Fibrosis

### 2.1. Histone Acetyltransferases (HATs) Role in Liver Fibrosis

The role of HATs in HSCs activation is not fully understood. The best characterized HATs are p300 and CREB binding protein (CBP), which are crucial regulators of liver fibrosis acting as transcriptional coactivators as well as acetyltransferases [70]. These HATs are able to acetylate not only histones but also transcription factors, leading to an increase in their binding to DNA [71]. It has been demonstrated that p300 mediates the TGFβ-induced expression of collagen in fibroblasts [72]. Indeed, CBP and p300 promote the acetylation of the N-terminal region of the SMAD2/3 via physical interaction, enhancing the transcription of their target genes [73]. In line with this, AMPK-induced proteasomal degradation of p300 attenuates SMAD3 activity in LX2 cells [74]. As aforementioned, Wnt/β-catenin pathway has a critical role in the activation of HSCs. Following its translocation to the nucleus, β-catenin associates with p300 and CBP to stimulate the transcription of its target genes [75]. Accordingly, pharmacological inhibition of the CBP/β-catenin complex blocks activation of primary mouse HSCs and enhances fibrosis resolution through the upregulation of matrix metalloproteinases (MMPs) expression in liver tissues from CCl_4_ mouse model of liver fibrosis [75]. Finally, p300 has a crucial role in the mechanical activation of HSCs in culture in response to surface stiffness [76]. Stiffness promotes p300 phosphorylation and translocation to the nucleus in parallel with increased protein levels of α-SMA and CTGF in primary human HSCs and LX2 cells. Consistently, shRNA-mediated knockdown of p300 suppresses activation of primary cultured mouse and human HSCs [76]. Even though emerging evidences highlight the role of HATs in HSCs activation and may represent novel targets for suppressing liver fibrogenesis, this field is out of the scope of this review. The reader is referred to recently published relevant articles [70,77].

### 2.2. Class I, II, and IV HDACs Role in Liver Fibrosis

There are numerous studies highlighting the potential role of HDACs in liver fibrosis. HDAC1 and HDAC2 are upregulated during the early stages of primary mouse HSCs activation in culture, although their expression was reduced afterwards [78]. This study also demonstrated the upregulation of HDAC8 expression at later time points of HSC activation in culture. Importantly, siRNA-mediated knockdown of class I HDACs decreased *LOX* mRNA levels [78]. Moreover, a study evaluating class II HDACs role in HSC transdifferentiation described that HDAC9 and 10 expression is downregulated in this process, while HDAC4 and 7 remain constantly expressed [79]. However, the changes in mRNA levels of specific HDACs during hepatic fibrogenesis appear complex. Indeed, a recent study demonstrated that CCl_4_-induced liver fibrosis promoted the upregulation of several HDAC mRNAs, while fibrosis reversal was accompanied by the downregulation of the expression of specific HDACs [80]. Whereas most HDACs were upregulated during fibrosis (HDAC1, 2, 4, 5, 6, 8, 9, and 10), only HDAC2, 6, and 8 were downregulated during resolution of fibrosis, in parallel with the upregulation of HDAC11. In fact, the authors reported increased expression of HDAC2 in HSC-T6 cells upon treatment with TGFβ1 as well as in human fibrotic liver tissue [80]. In agreement with this study, upregulation of HDAC2, 6 and 8 in a CCl_4_ in vivo model as well as in TGFβ-stimulated LX2 cells (a human cell line of HSCs) has been documented [81], observing no changes in class I HDAC protein levels [82]. HDAC9 expression is also markedly higher in diseased human livers, including primary biliary cirrhosis (PBC), alcoholic cirrhosis and NASH [83]. These differences between studies could be model-dependent, thus further research is needed to establish global HDAC expression changes during hepatic fibrogenesis.

**Figure 1:**
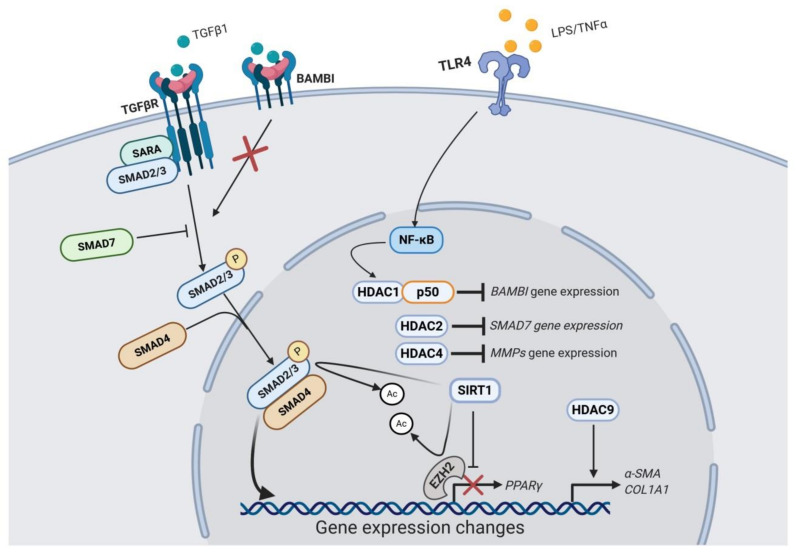
Schematic representation of the role of the best-characterized HDACs in liver fibrosis. TGFβR activation induces the phosphorylation of the SMAD2/SMAD3 complex, which binds to SMAD4 to translocate to the nucleus and regulates gene expression. Cellular responses to TGFβ are modulated in part by HDACs. HDAC2 inhibits the expression of SMAD7, a negative regulator of TGFβ signaling. HDAC4 inhibits the expression of MMPs genes, and HDAC9 appears to be involved in the expression of *α-SMA* and *COL1A1* genes. The activation of HSCs can be enhanced by NFκB-mediated signaling, which promotes the formation of the HDAC1-p50 complexes. This complex inhibits the expression of BAMBI, which is a negative regulator of the TGFβ pathway. On the contrary, SIRT1 has an antifibrogenic role through the inhibition of the SMAD2/SMAD3/SMAD4 complex and the HMT EZH2 via lysine deacetylation.

Even though HDACs expression changes and their significance are poorly understood, a role for various individual HDACs in liver fibrosis has been proposed (Figure 1). HDAC1 is part of the NF-κB-HDAC1 complex that enhances TGFβ signalling through transcriptional repression of the *BAMBI* gene promoter, a negative regulator of the TGFβ signaling [84]. Consistently, HDAC1 depletion blocked LPS/TNFα-induced *BAMBI* downregulation in LX2 cells [84]. These results highlight the importance of injury-associated inflammation on the fibrogenic response. HDAC2 seems to participate in the suppression of *SMAD7* gene expression, a negative regulator of the TGFβ signaling pathway [80]. HDAC4 upregulation is involved in histone deacetylation of the MMPs genes promoters, which blocks the recruitment of transcription factors such as c-Jun and p65 and leads to the epigenetic repression in the MMP genes at the chromatin level. As a result, ECM turnover is impaired, perpetuating the accumulation of fibrotic tissue [82]. According to this, ectopic expression of HDAC4 in quiescent HSCs suppresses the MMPs promoter transcriptional activity [82]. In quiescent HSCs, HDAC4 downregulation is carried out by cathepsin-H, which is involved in the HDAC4 protein digestion, allowing a permissive epigenetic state for MMPs expression under liver injury [85]. Moreover, protein levels of several cysteine cathepsins were progressively downregulated during HSC transdifferentiation [85]. These authors further showed that in human cirrhotic liver cathepsin-H expression is mostly absent in parallel with high levels of HDAC4 in the fibrotic septa [85]. Furthermore, miR-29a-mediated inhibition of HDAC4 suppresses the profibrogenic phenotype of HSCs. Indeed, overexpression of miR-29a reduced HDAC4 expression and its nuclear translocation in vivo and in vitro in parallel with increased expression of H3K9Ac levels [41]. In addition, siRNA-mediated HDAC4 knockdown upregulates miR-29 expression [79]. HDAC9 repression interferes with TGFβ-target genes expression, such as α-SMA and *COL1A1*, as demonstrated in LX2 cells transfected with HDAC9 siRNA [83]. Even though it seems that HDAC4 has a crucial role in hepatic fibrogenesis, its enzymatic activity requires the association with HDAC3 through a corepressor complex called SMRT/N-CoR [86]. Therefore, it is important to highlight that HDACs can be functionally relevant either by their enzymatic activity or by their structural interaction with other complexes.

### 2.3. Class III HDACs (Sirtuins) Role in Liver Fibrosis

Sirtuins are HDACs involved in the deacetylation of a wide range of targets including not just histones, but also transcriptional regulators, in the nucleus. However, sirtuins have also been demonstrated to deacetylate proteins in other cellular compartments such as cytoplasm and mitochondria [87]. Although they are known regulators of caloric restriction beneficial effects, sirtuins have also been linked to cell survival and apoptosis in cancer, DNA repair, development, inflammation and neuroprotection [88]. Recently, sirtuins have been proposed as new therapeutic targets for the treatment of liver fibrosis (Table 2), being SIRT1 the most well-characterized (Figure 1) [89]. SIRT1 is downregulated during HSCs activation as demonstrated in in vitro studies, including TGFβ-stimulated LX2 cells [90,91], and activated rat [92] and mouse [90,93] primary HSCs. It has also been found downregulated in fibrotic liver tissues from in vivo studies in models such as CCl_4_ [90,93,94], thioacetamide (TAA) [93] and ethanol-induced liver injury in mice [95], and CCl_4_ [92] and BDL rat models [96,97], as well as in non-alcoholic fatty liver disease (NAFLD) patients liver tissues [98]. SIRT1 downregulation in hepatic fibrogenesis seems to be modulated in part by epigenetic mechanisms, including HDAC4 and lncRNA MALAT1-mediated SIRT1 repression [90,93]. It can also be regulated by other proteins such as Protein Inhibitor of Activated STAT4 (PIAS4), which is upregulated in steatosis-associated liver fibrosis and mediates SIRT1 transcriptional repression in primary mouse HSCs [99]. Consistently, SIRT1 protein expression is restored during fibrosis reversal in parallel with *α-SMA* downregulation [90]. Moreover, SIRT1 overexpression induces apoptosis and blocks proliferation of TGFβ-activated LX2 cells [90]. Indeed, in HSC-specific SIRT1 knockout mice CCl_4_-induced liver fibrosis is exacerbated [93].

Mechanistically, SIRT1 seems to be involved in hepatic fibrogenesis inhibition through different pathways. First, SIRT1 potentiates *PPARγ* activity via deacetylation, which antagonizes HSC activation. According to this, *PPARγ* inhibition blocks the antifibrogenic effects of SIRT1 overexpression in HSCs [93]. Second, SIRT1-mediated SMAD3 deacetylation blocks TGFβ-induced LX2 and mouse primary HSCs activation [89,99]. Third, SIRT1-mediated deacetylation of EZH2, which is involved in HSCs transdifferentiation to myofibroblasts, decreases its stability, therefore facilitating its degradation in LX2 and rat primary HSCs exposed to TGFβ [92]. Finally, SIRT1 also participates in the activation of the liver kinase B1 (LKB1)/AMP-activated protein kinase (AMPK) pathway, which is suggested as a potential target for liver protection [100,101,102]. Indeed, pharmacological activation of AMPK has been demonstrated to inhibit TGFβ-induced intracellular lipid droplets depletion and activation of LX2 cells [103]. Moreover, AMPK activation has also been shown to attenuate liver injury and fibrosis in BDL rats through non-canonical NF-κB pathway inhibition and subsequent downregulation of inflammatory cytokines such as *Tnf-α*, *Il-β*, *Il-21*, and *Ccl21* [104]. However, a recent study suggested that TWEAK-induced SIRT1 upregulation inhibits LX2 cells senescence in parallel with α-SMA increased expression [105]. Therefore, more studies are required to fully understand the potential roles of SIRT1 in the hepatic fibrogenesis.

Even though SIRT1 is the most characterized member of this family in liver fibrosis, there are others sirtuins involved in the hepatic fibrogenesis. For instance, SIRT2 is overexpressed in human fibrotic liver tissues [106]. Pharmacological or genetic (shRNA) SIRT2 inhibition suppresses fibrogenic gene expression in LX2 cells and mouse primary HSCs [106]. Indeed, SIRT2 overexpression increased *α-Sma* and *Cola1* gene expression in *Sirt2*-KO mouse primary HSCs. *Sirt2*-KO mice display reduced hepatic fibrogenesis after CCl_4_ and TAA treatment [106]. SIRT2 seems to be involved in the activation of the ERK pathway through deacetylation of the ERK protein, which, in the end, promotes fibrogenesis via inhibition of c-MYC protein degradation [106,107]. An antifibrogenic role for SIRT3 has been established. SIRT3 is downregulated in primary rat HSCs and rat CCl_4_-induced fibrosis [108]. *Sirt3*-KO mice develop tissue fibrosis in multiple organs including lung, kidney and liver with age [109]. Consistently, *Sirt3*-overexpressing transgenic mice have attenuated liver fibrosis associated with reduced SMAD3 protein levels in liver tissues, therefore SIRT3 plays a role in regulating TGFβ signaling [109]. According to this, four pathways for SIRT3 antifibrotic function have been proposed [110]. It is known that reactive oxygen species (ROS) induce HSCs transdifferentiation and stimulate ECM production [111]. In line with this, SIRT3 potentiates superoxide dismutase 2 (SOD2) activity via its deacetylation, which prevents the accumulation of ROS in LX2 and HSC-T6 cells [110]. In addition, SIRT3 also reduces intracellular oxidative stress in LX2 cells through inhibition of NOX (NADPH oxidase) activity, family of enzymes responsible for the generation of superoxide and hydrogen peroxide [112]. SIRT3 interferes in the TGFβ pathway in HSCs through the degradation of SMAD proteins via activation of GSK-3β (Glycogen synthase kinase 3 beta). SIRT3-mediated GSK-3β activation induces β-catenin phosphorylation and degradation, which is crucial to prevent HSCs transdifferentiation [63,110] and, in part mediates the antifibrogenic effects of the activation of AMPK signalling. Indeed, siRNA-mediated AMPK silencing suppresses SIRT3 expression [108]. It has been shown that SIRT4 is downregulated in human fibrotic liver tissues [113], although little is known about the specific role in liver fibrosis. Finally, an early study showed that *Sirt6*-KO mice exhibited increased collagen deposition in the liver [114]. Recently, this appreciation was confirmed by a study that showed exacerbated fibrosis in *Sirt6*-KO mice while *SIRT6*-overexpressing transgenic mice were protected against fibrosis [115]. Moreover, SIRT6 levels were considerably reduced in patients as fibrosis progressed to cirrhosis [115]. In addition, SIRT6 was also downregulated in culture-activated mouse primary HSCs and TGFβ-treated mouse primary HSCs. In fact, shRNA-mediated SIRT6 silencing enhances the expression of profibrogenic markers such as *COL1A1*, *ACTA2*, and *TIMP1* in TGFβ-stimulated LX2 cells [115]. Mechanistically, SIRT6 seems to have a dual function, it can deacetylate SMAD3 proteins and inhibit their transcriptional activity on target genes, and it can also deacetylate ac-H3K9 residues in SMAD3 target genes promoters such as *COL1A2* and *TGFβ1* [115].

## 3. HDAC Inhibitors as Therapeutic Tools to Treat Liver Fibrosis

HDAC inhibitors (HDACIs) are compounds that are able to chelate the HDACs Zn^2+^ ion, which is fundamental for their deacetylase activity [116]. HDACIs are generally classified into four classes: short chain fatty acids (SCFAs), hydroxamic acids, benzamides and cyclic peptides (Table 3) [29]. However, we will describe them according to their specificity for the different HDACs. Even though recent studies also provide evidence of the therapeutic potential of SIRTs targeting, there are no compelling publications using specific SIRT inhibitors in hepatic fibrogenesis yet.

### 3.1. Pan-HDAC Inhibitors

**Figure 2:**
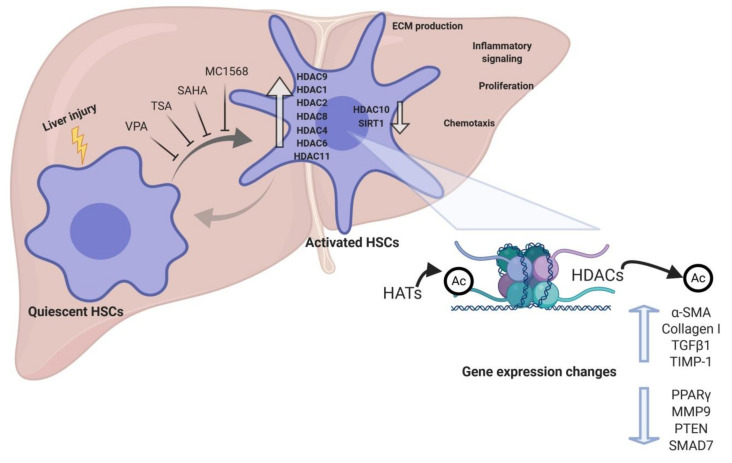
HDACs gene expression changes during activation of HSCs. Liver injury induces the activation of HSCs, which is characterized by increased production of ECM components and inflammatory mediators, as well as by increased proliferation and chemotaxis. During HSC activation several HDACs are differentially expressed, and their activity contributes to the expression of antifibrogenic as well as profibrogenic genes. HDAC inhibitors such as VPA, TSA, SAHA, and MC1568 inhibit the transdifferentiation of quiescent HSCs into myofibroblast-like fibrogenic HSCs.

Pan-HDAC inhibitors are small molecules able to inhibit class I and II HDACs activity. The first study using HDACIs for the treatment of hepatic fibrogenesis was published in 1999 [117]. The aim of this study was to compare the antifibrogenic effect of two HDACIs: sodium butyrate, a non-competitive class I and IIa inhibitor that requires millimolar concentrations, and trichostatin A (TSA), which is a reversible class I and II inhibitor and effective at submicromolar concentrations (Figure 2). While sodium butyrate showed a modest inhibition of profibrogenic genes expression, TSA significantly suppressed activation, proliferation, and transdifferentiation of rat primary HSCs, followed by an increase in overall H4 acetylation levels and affecting the development of the actin cytoskeleton [117,118]. TSA has also been shown to hamper the EMT-induced fibrogenic activation of AML12 mouse hepatocytes and human primary hepatocytes, exerting its antifibrogenic effects by preventing the SMAD3-SMAD4 complex formation and interfering with p300 activity within this complex at the collagen gene promoter [119]. TSA suppresses hepatic fibrogenesis in the in vivo CCl_4_ mouse model, as well as in HSC-T6, LX2 and rat primary HSC cells, through the stabilization of C/EBP-α protein via its acetylation, being C/EBP-α a known negative regulator of HSC activation [120,121]. Even though all these evidences support TSA as an antifibrogenic therapy for liver fibrosis, TSA can be metabolized within 30 min by the hepatocytes [122], therefore, other pan-HDACIs with improved efficiency, safety, and pharmacokinetics have been produced. Park and colleagues developed a new HDACI called N-hydroxy-7-(2-naphthylthio)heptanomide (HNHA), a class I and II HDACI, that showed strong properties against liver fibrosis [123]. HNHA induces the upregulation of cell cycle arrest genes such as p21 and p53 and pro-apoptotic (Bad, Bax, and Bak) members of the Bcl-2 family as well as several caspases, which are major effectors in apoptosis [123]. Consequently, it suppressed cell proliferation and induced apoptosis of PDGF-activated mouse and human primary HSCs. Moreover, it repressed the protein expression of profibrogenic genes such as COL1A1, α-SMA, TGFβ1, and MMP2/9 in PDGF-induced mouse primary HSCs and liver tissues from BDL mouse model. Interestingly, HNHA also exerted hepatoprotective effects in parallel with a reduction in the levels of circulating transaminases (ALT and AST) and total bilirubin (Tbil) [123]. Suberoylanilide hydroxamic acid (SAHA), a class I and II HDAC inhibitor, is an analog of TSA and has already been demonstrated to suppress human HSCs activation [123]. SAHA, also called vorinostat, was the first HDACI approved by the Food and Drug Administration (FDA) in the United States for the treatment of cutaneous T-cell lymphoma (CTCL) [124]. It has been demonstrated that SAHA is able to attenuate HSCs activation accompanied by a reduction in the expression of α-SMA and collagen I in LX2 cells. Furthermore, SAHA-induced suppression of HSC activation is in part mediated by NF-κB downregulation, which plays a crucial role in liver fibrosis [125,126], through the acetylation of high mobility group box 1 (HMGB1) [125]. SAHA also alleviates liver fibrosis by suppressing TGFβ signalling via phospho-SMAD2/3 reduction and SMAD7 upregulation in LX2 cells and mouse liver tissues from the CCl_4_ model. This was accompanied by downregulation of α-SMA, collagen, and CTGF protein expression, and increased H3 acetylation levels [81]. We have to take into account that inhibiting all HDACs may carry undesired side effects. Consequently, more selective HDACIs that can overcome this toxicity are being developed.

### 3.2. Class-Specific HDAC Inhibitors

This group of HDACIs includes molecules that are able to inhibit more efficiently specific HDACs. Even though valproate (VPA) and its sodium salt, sodium valproate, can inhibit class II HDACs, they also strongly suppress class I HDACIs [29,127]. Sodium valproate has been well established as a long-term therapy of epilepsy, which attests to the safety of this molecule when chronically administered in humans, at least in patients without preexisting liver conditions [127,128]. However, it has also been proposed as a treatment for liver fibrosis by several experimental studies, therefore expanding its clinical applications (Figure 2). Mannaerts and colleagues demonstrated that VPA chronic treatment attenuates CCl_4_-induced fibrosis in mice as shown by reduced collagen deposition and *Acta2, Col1a1, Timp-1*, and *Mmp13* downregulation. Moreover, the authors show that VPA inhibits mouse primary HSCs activation [78]. Indeed, VPA treatment blocks the TGFβ1 autocrine loop and attenuates TGFβ-induced collagen synthesis in a human HSC line via reduction of SMAD2/SMAD3 phosphorylation, which was paralleled by increased H3 and H4 acetylation levels [129]. VPA has also been shown to induce apoptosis in activated HSCs and to reduce α-SMA protein levels as well as collagen deposition in the TAA-induced fibrosis mouse model. Furthermore, this study also revealed a hepatoprotective role for VPA in TAA-induced liver damage, as its administration decreased circulating ALT and AST levels [130]. VPA is able to significantly reduce *S. mansoni*-induced fibrosis and inflammation through downregulation of profibrogenic factors and collagen deposition [131]. VPA antifibrogenic properties were corroborated in an in vivo rat model of CCl_4_-induced fibrosis, which were characterized by *α-Sma* downregulation, circulating AST and ALT reduction and decreased collagen deposition [127]. Although compelling evidences support the promising effects of HDACIs in liver fibrosis, the underlying mechanisms are still not well known. However, a study published in 2019 proposed that VPA modulated the expression of several miRNAs involved in hepatic fibrogenesis, suggesting a strong crosstalk between histone acetylation and miRNAs expression in the activation of HSCs [132]. In addition to VPA, there are other HDACIs that display even more selectivity. Largazole, a natural compound isolated from the marine cyanobacterium *Symploca sp.* [133], has been proved to inhibit mouse and human primary HSCs activation, a response which was associated with increased H3 and H4 acetylation levels. Accordingly, largazole represses the expression of profibrogenic markers and effectors such as α-SMA, collagen I, and TIMP-1 [134]. This HDACI also attenuates hepatic fibrogenesis in the in vivo mouse model of CCl_4_-induced liver fibrosis. The antifibrogenic in vivo effects of largazole were characterized by increased HSCs apoptosis, reduced levels of circulating liver enzymes (AST, ALT and ALP), and α-SMA, collagen I and TIMP-1 downregulation. Mechanistically, largazole inhibited TGFβR2 expression, as well as TGFβ1-induced phosphorylation of SMAD2 and AKT [134]. Apart from TGFβ1-induced signaling, vascular endothelial growth factor (VEGF) also contributes to HSC activation [135,136]. In line with this, largazole inhibited VEGF-induced signalling pathway via suppression of AKT and p38 phosphorylation [134]. Finally, a very recent study using five more selective HDACIs revealed that inhibitors of class I HDACs, but not class II enzymes, attenuate TAA-induced liver fibrosis in mice by suppressing hepatic type 2 inflammation [137]. Type 2 inflammation exerts many protective functions such as maintenance of barrier defence and regulation of tissue regeneration [138], and is characterized by increased production of IL-4, IL-5, IL-9, and IL-13 cytokines [137,138]. These cytokines are produced by recruited inflammatory cells, including eosinophils, basophils, macrophages, and group 2 innate lymphoid cells (ILC2), which are also an important source [138]. However, several diseases such as primary sclerosing cholangitis, primary biliary cirrhosis and NASH involve chronic activation of type 2 response, which may lead to fibrosis [138]. Indeed, this study also showed that class I HDAC inhibition reduces HSCs activation in liver tissues as demonstrated by downregulated *Acta2* expression and α-SMA protein levels. Furthermore, authors suggest a critical role for the HDAC1 and HDAC2 in the antifibrogenic activity of class I HDAC inhibitors [137]. Despite of the fact that further studies are needed to evaluate the efficacy of these selective class I HDACIs in other models of hepatic fibrogenesis, these evidences underscore the potential of class I HDAC inhibition as a therapy for liver fibrosis.

Unfortunately, HDACIs have been shown to carry side effects such as nausea, vomiting, fever, and headache, which can be easily managed [139]. However, serious adverse effects, such as hepatotoxicity, have also been observed [140], including a clinical case of steatohepatitis and hepatocellular carcinoma [141]. VPA has been associated with dose-dependent elevation of serum aminotransferases [142]. More importantly, long term VPA-therapy is linked to weight gain which may lead to insulin resistance and NAFLD [143]. It is necessary to be prudent with the use of VPA as clinical treatment for liver fibrosis [144]. Indeed, the currently approved five HDACIs (belinostat, chidamide, panobinostat, romidepsin, and vorinostat) are not recommended in patients with severe hepatic dysfunction [139]. Furthermore, the effects of HDACIs are likely to be genome-wide. Hence, the side effects are probably different depending on the genetic background as well as on the age of the patient. [139]. Therefore, the clinical utility of these HDACIs in liver fibrosis is limited due to the ratio risks/benefits. Consequently, several HDACIs with higher selectivity and reduced inhibition of class I HDACs are being developed in order to reduce the risk of side effects. As discussed below, the targeted delivery of HDACIs to activated liver stellate cell could also circumvent in part their hepatocellular and systemic toxicity.

Although it was previously mentioned that class II HDAC inhibitors do not exhibit antifibrotic effects in TAA fibrosis model, a study carried out by Mannaerts and colleagues demonstrated that class II HDAC inhibition blocked mouse primary HSCs activation [79]. The authors highlight that class II HDAC inhibition would be preferred due to the fact that class II HDACs are not generally involved in the control of gene transcription and, therefore, they would be less toxic [79]. MC1568, a selective class II HDACI, markedly decreases mouse primary HSCs activation in parallel with downregulation of profibrogenic genes such as *Col1a1*, *Col3a1*, *Acta2*, and *Lox*. This effect was mediated by the upregulation of miR-29 expression, which is a known antifibrotic miRNA [79], further indicating the importance of the crosstalk between epigenetic modulations and the necessary search towards more selective effects.

## 4. Conclusions

Emerging experimental evidence indicates the importance of epigenetic remodeling in the activation of HSCs and liver fibrogenesis. Several studies demonstrate that HDACs are crucial regulators of the activation of HSCs, showing important changes in expression during liver fibrosis (Figure 2). Indeed, in vitro and in vivo genetic manipulation of HDACs has been shown to affect HSC transdifferentiation to myofibroblasts, and experimental studies using HDACI have shown remarkable results in reducing liver fibrosis. Nevertheless, given the complexity of epigenetic mechanisms, including the crosstalk between different epigenetic events in gene regulation, further studies are required to dissect their role in HSC activation. It is possible that adverse effects to “epidrugs” such as HDACI may emerge due to their pleiotropic effects on gene expression and their activity on non-histone targets. Another aspect that needs to be addressed is the specific targeting of HDACI to HSCs. This is an important issue when drugs are administered systemically, and is particularly relevant in patients with impaired liver function. In this regard, different strategies in drug formulation are being explored for HSC-targeted delivery of epigenetic drugs [60,145]. In spite of these potential limitations, this field holds promise for the development of new therapies for liver fibrosis and, thus, improve the outcome of patients with CLD.

## Figures and Tables

**Table 1:** Classification and subcellular localization of HDACs.

HDAC Class	Subcellular Localization	HDACs
Class I HDACs (Zn^2+^-dependent HDACs)	Nucleus	HDAC1
		HDAC2
		HDAC3
		HDAC8
Class IIa HDACs (Zn^2+^-dependent HDACs, one catalytic site)	Cytoplasm/Nucleus	HDAC4
		HDAC5
		HDAC7
		HDAC9
Class IIb HDACs (Zn^2+^-dependent HDACs, two catalytic sites)	Cytoplasm/Nucleus	HDAC6
		HDAC10
Class III HDACs (SIRTs) (NAD^+^-dependent HDACs)	Nucleus/Cytoplasm	SIRT1, SIRT2
	Nucleus/Mitochondria	SIRT3
	Mitochondria	SIRT4, SIRT5
	Nucleus	SIRT6
	Nucleolus	SIRT7
Class IV HDACs (Zn^2+^-dependent HDACs)	Nucleus	HDAC11

**Table 2:** HDACs expression in experimental models of liver fibrosis.

Expression	HDAC	Experimental Model
Increased expression	HDAC1	Primary mouse HSCs [78], mouse CCl_4_-fibrosis [80]
	HDAC2	Primary mouse HSCs [78], mouse [80] and rat [81] CCl_4_-fibrosis, TGFβ-treated HSC-T6 [80] and LX2 cell lines [81], human fibrotic liver [80]
	HDAC8	Primary mouse HSCs [78], rat CCl_4_-fibrosis, TGFβ-treated LX2 cells [81]
	HDAC4	Rat CCl_4_-fibrosis [80,82], primary rat HSCs [82]
	HDAC5	Mouse CCl_4_-fibrosis [80]
	HDAC9	Mouse CCl_4_-fibrosis [80], human PBC/alcoholic cirrhosis [83]
	HDAC6	Mouse [80] and rat [81] CCl_4_-fibrosis, TGFβ-treated LX2 cells [81]
	SIRT2	Human fibrotic liver tissues [106]
	HDAC11	Mouse CCl_4_-fibrosis [80]
Decreased expression	HDAC9	Primary mouse HSCs [79]
	HDAC10	Primary mouse HSCs [79]
	SIRT1	Primary mouse [90,93] and rat [92] HSCs, TGFβ-treated LX2 cells [90,91], mouse [90,93,94] and rat [92] CCl_4_-fibrosis, mouse TAA-fibrosis [93], mouse ethanol-fibrosis [95], rat BDL-fibrosis [96,97], NAFLD patients [98]
	SIRT3	Primary rat HSCs [108] and rat CCl_4_-fibrosis [108]
	SIRT4	Human fibrotic liver tissues [113]
	SIRT6	Human cirrhotic liver tissues [115] and primary mouse HSCs [115]

**Table 3:** HDAC inhibitors used in liver fibrosis according to their chemical structure.

Chemical Structure	HDAC Inhibitor	Targets
Hydroxamic acids	Trichostatin A (TSA)	Class I, II and IV HDACs
	N-hydroxy-7-(2-naphthylthio)heptanomide (HNHA)	
	Suberoylanilide hydroxamic acid (SAHA)	
Short chain fatty acids	Butyric acid/sodium butyrate	Class I and IIa HDACs
	Valproic acid (VPA)/sodium valproate	
Others	Largazole	HDAC1, HDAC2 and HDAC3
	MC1568	Class II HDACs
